# Microstructure and Mechanical Properties of Spark Plasma Sintered CoCrFeNiNbX High-Entropy Alloys with Si Addition

**DOI:** 10.3390/ma16062491

**Published:** 2023-03-21

**Authors:** Miroslav Karlík, Filip Průša, Petr Kratochvíl, Hana Thürlová, Angelina Strakošová, Jaroslav Čech, Jiří Čapek, Marek Vronka, Marcello Cabibbo, Ondřej Ekrt

**Affiliations:** 1 Faculty of Nuclear Sciences and Physical Engineering, Czech Technical University in Prague, Trojanova 13, 120 00 Prague, Czech Republic; 2 Department of Metals and Corrosion Engineering, University of Chemistry and Technology, Technická 5, 166 28 Prague, Czech Republic; 3 Institute of Physics of the Czech Academy of Sciences, Na Slovance 1999/2, 182 21 Prague, Czech Republic; 4 Dipartimento di Ingegneria Industriale e Scienze Matematiche (DIISM), Università Politecnica delle Marche, Via Brecce Bianche 12, 60131 Ancona, Italy

**Keywords:** high-entropy alloys, mechanical alloying, spark plasma sintering, X-ray diffraction, electron microscopy, instrumented indentation, compression testing

## Abstract

Three mechanically alloyed (MA) and spark plasma sintered (SPS) CoCrFeNiNbX (X = 5, 20, and 35 at.%) alloys with an addition of 5 at.% of SiC were investigated. The face-centered cubic (FCC) high-entropy solid solution, NbC carbides, and hexagonal Laves phase already developed during MA. In addition, the SPS compacting led to the formation of oxide particles in all alloys, and the Cr_7_C_3_ carbides in the Nb5 alloy. The fraction of the FCC solid solution decreased with increasing Nb concentration at the expense of the NbC carbide and the Laves phase. Long-term annealing at 800 °C led to the disappearance of the Cr_7_C_3_ carbide in the Nb5 alloy, and new oxides—Ni_6_Nb_6_O, Cr_2_O_3_, and CrNbO_4_—were formed. At laboratory temperature, the Nb5 alloy, containing only the FCC matrix and carbide particles, was relatively strong and very ductile. At a higher Nb content (Nb20 and Nb35), the alloys became brittle. After annealing for 100 h at 800 °C, the Nb5 alloy conserved its plasticity and the Nb20 and Nb35 alloys maintained or even increased their brittleness. When tested at 800 °C, the Nb5 and Nb20 alloys deformed almost identically (CYS ~450 MPa, UTS ~500 MPa, plasticity ~18%), whereas the Nb35 alloy was much stronger (CYS of 1695 MPa, UCS of 1817 MPa) and preserved comparable plasticity.

## 1. Introduction

Since 2004, the research of high-entropy alloys (HEAs) has been steadily surprising the wide scientific community by constantly pushing the boundaries of achieved results. This is related to the development of new, advanced techniques that allow the preparation of alloys composed of elements with a high melting point. Usually, this is achieved by a high current density imposed over a small area of the material to be melted, referring to arc-melting techniques. Another approach is to use high-energy sources, which are commonly used in additive manufacturing. Both approaches allow for the preparation of miniaturized samples rapidly dissipating heat allowing them to be cooled down within a matter of seconds. This may be sufficient to suppress the formation of coarse-grained structures within the HEA, although the cooling rates are usually low in order to enhance the supersaturation of solid solutions, making the precipitation strengthening via a proper consequential heat treatment highly unlikely, but not entirely impossible. He et al. [1] reported precipitation of γ″ phase which is known as the most common strengthening phase in Ni superalloys—Inconel 706, 709, or 718. However, the γ″ phase undergoes a further peritectic transformation γ″ + face-centered cubic (FCC) matrix → ε. The hexagonal ε-(NiCo)_3_Nb phase is surprisingly formed instead of the δ-Ni_3_Nb phase commonly found in superalloys. Another major setback can be the porosity and overall structural heterogeneity of the additively manufactured samples causing high anisotropy of the properties related to the building strategy.

The CoCrFeNiMn alloy, also referred to as Cantor alloy, belongs to the most studied HEA, providing a variety of properties based on the chosen preparation technique. It is usually a single-phase alloy containing a disordered FCC solid solution. This phase composition might seem stable, but it creates Cr-enriched phases during long-term annealing [2], which are identified as σ secondary phases, known for degradation of their properties. On the other hand, this phase might be replaced by Cr-based carbides which tend to form preferentially in systems containing C. Nevertheless, the formation of carbides is less harmful compared to the σ phase. On the other hand, a similar effect can be achieved by changing the actual chemical composition of the Cantor alloy. Full substitution of the Mn by Nb is known for the creation of a two-phase structure composed of FCC solid solution and newly formed C14 Laves phase. The volume fraction of the Laves phase increases with the total content of Nb within the alloy, strengthening the alloy although at the expense of ductility [3,4,5]. Furthermore, the formation of the Laves phase is also preferred due to the highly negative mixing enthalpies with other elements [6]. In addition, as was already mentioned above, the CoCrFeNiNb alloys are known for the formation of precipitates when annealed at 750 °C, which opens a new promising method for further improving their properties.

Nowadays, CoCrFeNiNb high-entropy alloys are prepared using various processing technologies such as arc melting [5,6,7,8,9,10,11,12,13], arc melting accompanied by suction casting [14,15], additive manufacturing using direct laser deposition [16], and powder plasma arc additive manufacturing [17]. Although these processes might be beneficial for obtaining highly promising properties including complex shapes for the additively manufactured alloys, they often result in products composed of coarse-grained dendritic-like microstructures. Mechanical alloying is still a processing technology that remains rightfully standing among these techniques since it outperforms them in some respects. It is known to provide significant microstructural refinement while enhancing the supersaturation of present phases. In addition, the process is known for establishing an equilibrium between continuous plastic deformation and dynamic recovery and recrystallization. Therefore, mechanical alloying is capable of reaching the maxima properties throughout a variety of alloys.

This paper describes the influence of Nb content on the properties of mechanically alloyed CoCrFeNiNbX (X = 5, 20, and 35 at.%) alloy with Si addition (2 to 3 at.%) compacted via spark plasma sintering.

## 2. Materials and Methods

**Figure 1:**
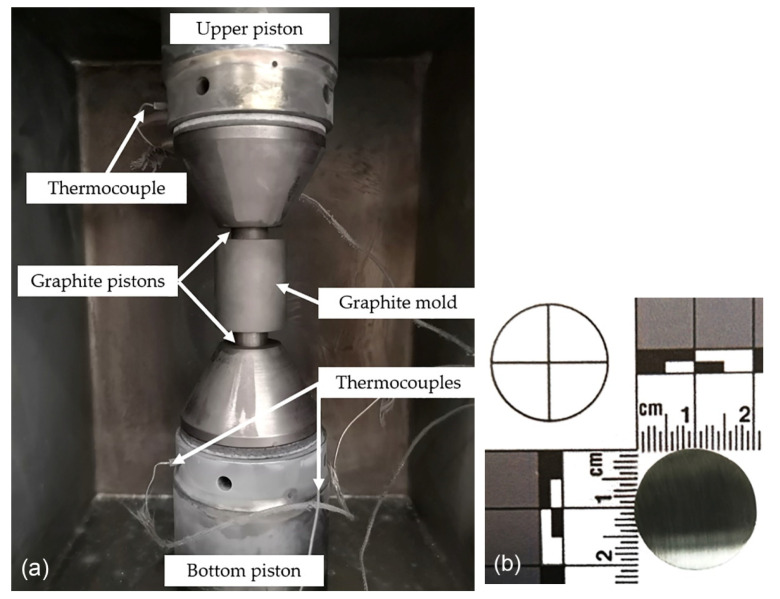
Chamber of the spark plasma sintering machine FCT Systeme HP-D10 (**a**), and a photo of one of the compacted samples (**b**).

The CoCrFeNiNbX (X = 5, 20, 35 at.%) high-entropy alloys (designated also as Nb5, Nb20, and Nb35) were prepared by mixing the pure elements of Co (2 μm, 99.8%), Cr (≤44 μm, ≥99%), Fe (5–9 μm, 99.9%), Ni (10 μm, 99.5%), and Nb (≤44 μm, 99.98%) in appropriate proportions to obtain 20 g powder batches. Moreover, 5 at.% of SiC (≤74 μm, ≥97.5%) was added to all of the mixtures to create particle-reinforced alloys. The mixtures were then charged in an AISI 420 stainless steel jar simultaneously with milling balls (weight ratio of ≈1:15) and 4 wt.% of n-heptane serving as process control agent (PCA), suitably reducing excessive cold welding. The mechanical alloying (MA) was realized using a Retsch PM 100 mill ran at 400 rpm in 30 min alloying segments alternated by 10 min cool-off breaks to achieve a total alloying period of 8 h. The MA powders were then compacted using a spark plasma sintering device FCT Systeme HP-D 10. For this purpose, 10 g of the powder was placed in a graphite mold (Figure 1a) and compacted using a heating rate of 200 °C/min up to the sintering temperature of 1000 °C, where the powder remained compressed for 10 min with a pressure of 48 MPa. Consequently, round samples 20 mm in diameter and ≈ 4 mm in height were obtained (Figure 1b).

The phase composition and the overall chemical composition of compact samples were determined using X-ray diffraction (XRD) (PANalytical X’Pert PRO MPD (Malvern Panalytical B.V., Almelo, The Netherlands), cobalt radiation (λ = 1.78901 × 10^−10^ m), and X-ray fluorescence (XRF) analysis (ARL XP 2400), respectively. The phase composition of annealed compacts was evaluated in the same XRD conditions using the Empyrean diffractometer (Malvern Panalytical B.V., Almelo, The Netherlands) equipped with the last generation high-energy resolution 1Der detector. The diffraction patterns were evaluated with X’Pert HighScore Plus, and crystallographic phases were identified using a PDF-2 database. Quantitative analysis was evaluated using the Rietveld analysis in the MStruct software [18]. The compacted samples were cut into several parts using Leco VariCut precise laboratory saw equipped with a diamond cutting blade. Standard metallographic cross-sections were examined using a light microscope (Nikon Eclipse MA 200, Yokohama, Japan). The surface porosity was determined before etching, which was conducted afterward using diluted aqua regia (mixture of HNO_3_ and HCl in the ratio of 1:3, further diluted 1:1 with distilled H_2_O). Porosity and area fractions of oxides/carbides on light microscopy (LM) or scanning (transmission) electron microscopy S(T)EM micrographs were evaluated using the threshold method in the ImageJ software.

The microstructure was examined using scanning electron microscopy (SEM, JSM IT500HR, JEOL, Tokyo, Japan, 10 kV), and scanning transmission electron microscopy (STEM, JEM-2200FS, JEOL, Tokyo, Japan, 200 kV) equipped with an energy dispersion spectrometer (EDS, Centurio, JEOL, Tokyo, Japan). A Ga+ focused ion beam (FIB, Quanta DualBeam, FEI, Hillsboro, OR, USA) was used for the preparation of STEM lamellae.

Instrumented indentation tests for hardness and indentation Young’s modulus determination were carried out on an MHT micro indentation tester (Anton Paar, Graz, Austria) equipped with a Vickers diamond tip. The maximum applied load was 1 N. Loading, holding at maximum load, and unloading were 30 s, 10 s, and 30 s, respectively. Data were analyzed by the Oliver-Pharr method [19] following ISO 14577 standard [20]. In addition, the thermal stability of prepared alloys was determined by the Vickers hardness HV 1 changes during long-term annealing at 800 °C. At least 10 measurements were performed to achieve good statistics; each imprint was positioned at least at a distance of three times greater than the diagonal of the imprint itself. The same rule was maintained when we approached the edges of the sample. A universal testing device LaborTech 5.250SP was used for compressives stress–strain testing on square prismatic samples with a height of 1.5× the size of the base, cut, and ground on a P800 abrasive paper. The tests were performed at the laboratory temperature in the sintered state, after 100 h of annealing at 800 °C, and also at an elevated temperature of 800 °C; the initial strain rate was 10^−3^ s^−1^.

## 3. Results and Discussion

### 3.1. Chemical and Phase Composition

#### 3.1.1. Powders and Compacts

To obtain alloys with high homogeneity, powders of high-purity elements were employed for mechanical alloying. From Table 1, it follows that the chemical composition of the compacts is somewhat shifted from the nominal one, especially in the case of iron, worn out from the stainless steel jar (differences from 2.76 up to 5.19 at.%). Other elements in the alloy almost have the targeted content; the maximum difference from the nominal composition is 2.68 at.% for cobalt in the Nb5 alloy; 8 other difference values over 12 are 1.52 at.% or lower. This is a relatively good match for the targeted composition. Furthermore, all the alloys also contain Si in the range from 2.21 to 3.06 at.%, corresponding to the addition of 5 at.% of SiC. The intention of alloying the alloys with SiC was to improve their mechanical properties due to a homogeneous distribution of ultrafine particles within the alloys. However, microstructural observations showed that the SiC underwent an in-situ reaction transforming into occasionally distributed particles of thermodynamically more stable SiO_2_ only in the Nb5 alloy, while in the Nb20 and Nb35 alloys, silicon started to dissolve in the present phases. The concentration of other elements in each of the alloys was under 0.1 at.%.

**Figure 2:**
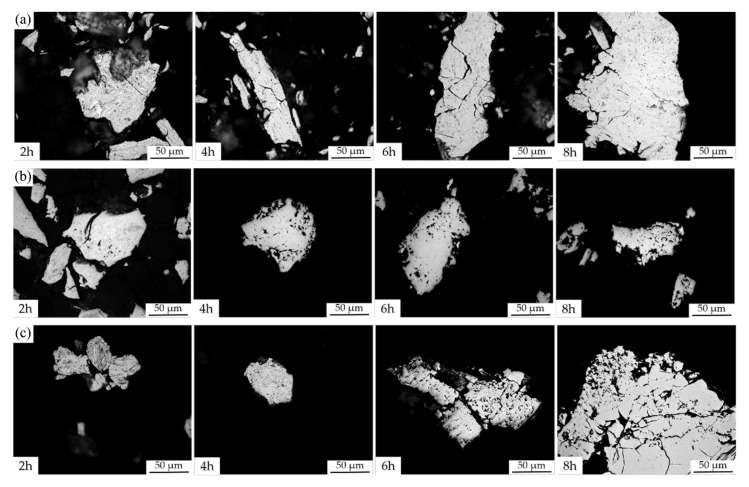
Light micrographs of the mechanically alloyed (MA) powders showing the evolution of the microstructure of powders of (**a**) CoCrFeNiNb5; (**b**) CoCrFeNiNb20; (**c**) CoCrFeNiNb35 alloy after mechanical alloying for 2, 4, 6, and 8 h.

**Figure 3:**
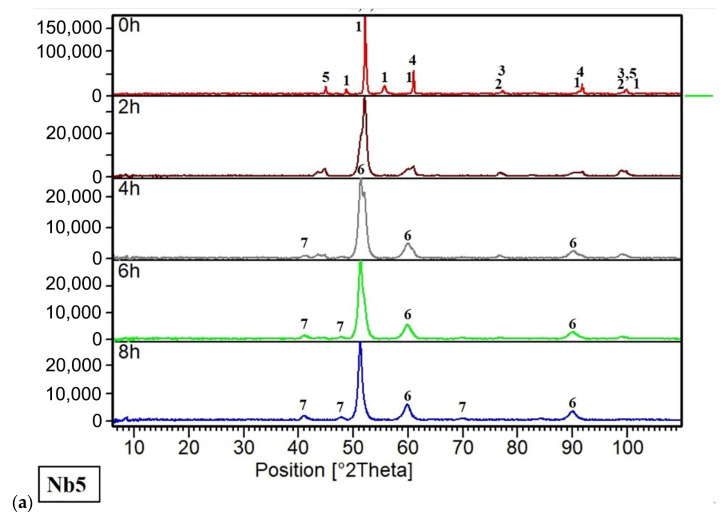

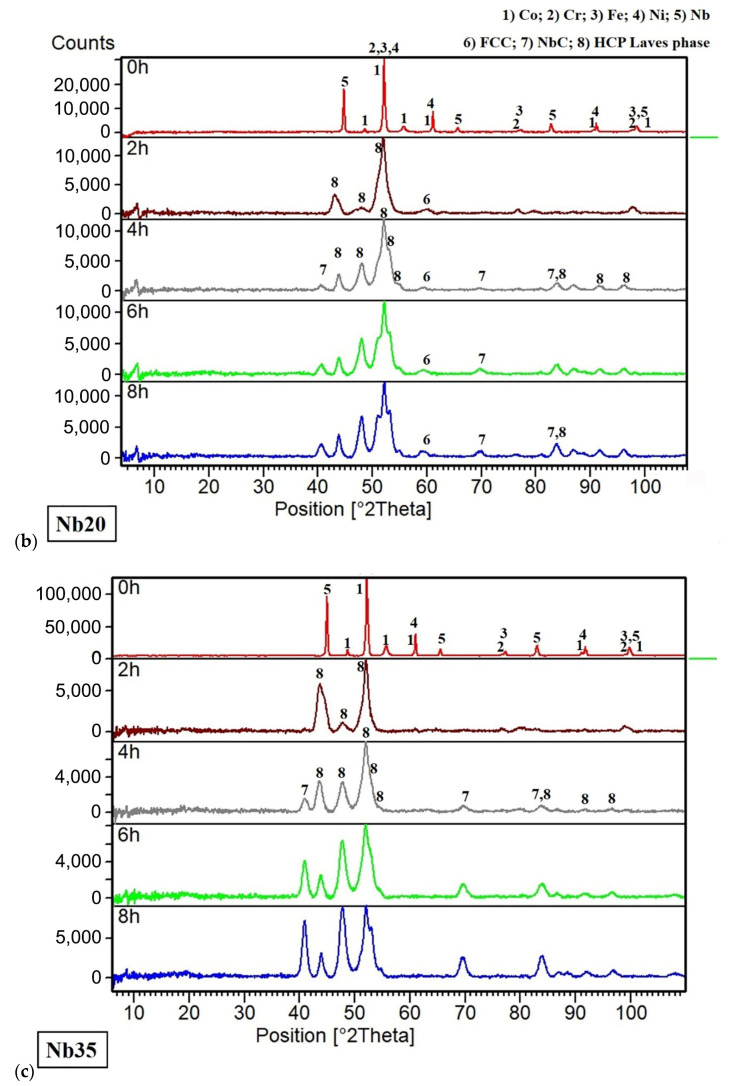
X-ray diffraction monitoring of the phase composition of powders during mechanical alloying: (**a**) CoCrFeNiNb5; (**b**) CoCrFeNiNb20; (**c**) CoCrFeNiNb35.

The evolution of the microstructure of the powders during mechanical alloying (MA) was monitored by light microscopy (Figure 2). After 2 h of MA, the powder particles of all alloys contained lamellae whose thickness was reduced over time. In addition, the alloys were showing certain brittleness manifesting itself by the presence of very fine particles and also by cracks, visible especially in the Nb35 alloy after 8 h of MA (Figure 2c). The phase development during MA was also examined by the XRD analysis (Figure 3) where a complete annihilation of the pure element reflections for the Nb20 and Nb35 alloys was observed after 6 h of the MA (Figure 3b,c), while the Nb5 alloy needed 8 h for the same result (Figure 3a). From this point of view, the higher the content of Nb, known as ductile material capable to achieve elongation reaching up to tens of % [21], the faster the phase’s creation.

This is related to the phenomena described in one of our former papers focused on Fe-Al-Si intermetallics [22], where we described the beneficial influence of the ductile phase on the incorporation of brittle powder particles within it intensifying the consequential processes of mutual diffusion and fragmentation, thus tremendously speeding up the whole process of alloying. Phases that have been formed during MA involve (1) a face-centered cubic (FCC) high-entropy alloy solid solution (also further abbreviated as FCC matrix), corresponding to the reference card of γ-Fe (Joint Committee on Powder Diffraction Standards (JCPDS) card no. 33-397, space group Fm3m); (2) NbC niobium carbide (JCPDS card no. 38-1364, space group Fm3m); (3) hexagonal close-packed (HCP) C14 Laves phase (JCPDS card no. 33-391, space group P6_3_/mmc).

**Figure 4:**
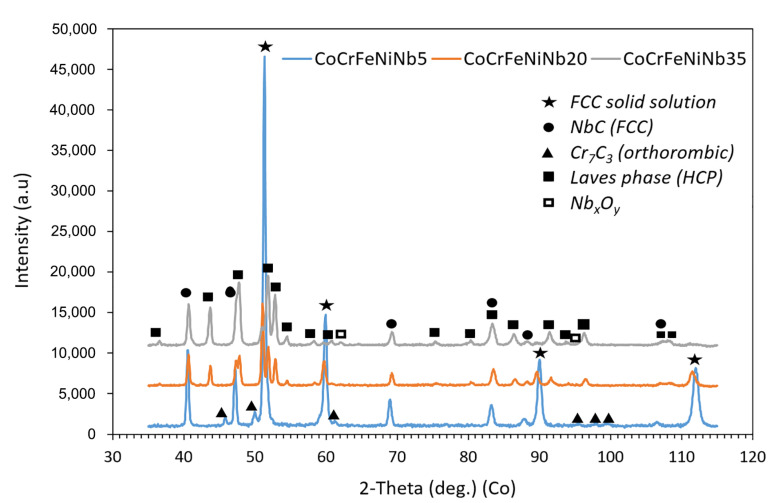
X-ray diffraction patterns of the CoCrFeNiNbX high-entropy alloy compacts prepared by mechanical alloying (MA) and spark plasma sintering (SPS).

The X-ray diffraction patterns of the compacted alloys are in Figure 4. Four phases containing the main alloying elements, and Nb_x_O_y_ oxides were identified (Figure 4, Table 2). In all alloys, an FCC solid solution, and NbC niobium carbide were found. The Nb5 alloy also contains Cr_7_C_3_ chromium carbide (JCPDS card no. 36-1482, space group Pmcn). On the other hand, in the alloys with higher niobium content (Nb20 and Nb35), an HCP C14 Laves phase, and a small amount (~1 wt.%) of Nb_x_O_y_ oxides formed (Table 2). Unambiguous identification of the type of Nb oxide (NbO_2_ or Nb_2_O_5_) is not possible, because X-ray analysis for such a low phase content is not sufficiently sensitive.

The presence of the HCP C14 Laves phase has also been reported in the literature using a variety of preparation techniques such as arc melting [5,6,7,8,9,10,11,12], arc melting and suction casting [14,15], additive manufacturing [16], and powder plasma arc additive manufacturing [17], or the phase composition was theoretically calculated using artificial neural network [13]. Among these phases, the presence of carbides (Cr_7_C_3_ or NbC), the weight fraction of which is way more important than those of NbxOy oxides, is the main difference from the above-mentioned methods. It is obvious that mechanical alloying is responsible for the supersaturation of the present phases with C, allowing the formation of carbides during the consequential SPS compaction. However, this cannot be considered a disadvantage since the significant microstructural refinement increases the mechanical performance of the alloy system.

From the Rietveld refinement analysis (Table 2), it follows that the fraction of the FCC solid solution decreases with increasing Nb concentration (from 75.7 to 51.9, and 7.4 wt.%) at the expense of the NbC carbide (9.8, 12.6, and 17.4 wt.%) and the Laves phase (0, 34.5, and 74.5 wt.%). The increasing amount of Laves phase with the Nb content has also been reported in the work of Liu et al. [5] describing the properties of arc-melted CoCrFeNiNbX (X = 0–10 at.%) alloy. The amount of Nb_x_O_y_ in Nb20 and Nb35 alloys is the same within the experimental error (1.0 and 0.9 wt.%). The Nb5 alloy also contains 14.5 wt.% of Cr_7_C_3_. The quality of the Rietveld method procedure was controlled by the weighted profile R-factor (R_wp_) figure of merit, which was 3.22–4.33%.

The changes in the crystal lattice parameters of the FCC matrix and NbC carbide with the increasing Nb content are not very pronounced—they are of the order of 0.4% (Table 3). The lattice parameter of the FCC solid solution increases in the Nb20 alloy and then slightly decreases in the Nb35 alloy. On the other hand, the NbC has the highest lattice parameter in the Nb5 alloy (0.44689 nm) and the lowest in the Nb35 alloy (0.44503 nm). The changes in the crystal lattice of the Laves phase with Nb content are also very small (Table 3); the values for the Nb20 alloy slightly differ from the Laves phase lattice (*a* = 0.4835 nm and *c* = 0.7860 nm) in the CoCrFeNiNb20 alloy previously prepared by Průša et al. [23] in similar conditions.

The spark plasma sintering (SPS) compaction reduced the internal porosity of all the investigated MA + SPS alloys (Table 4). The porosity of the Nb5 alloy was very small (0.03%) and it slightly increased with the Nb content to 0.23% for the Nb20 alloy and then remained almost the same for the Nb 35 alloy (0.25%). The increase in porosity can be attributed to the increasing fraction of the Laves phase (Table 2). The Nb5 alloy, composed mainly of the soft FCC solid solution, was subjected to intense plastic deformation during SPS compaction. On the other hand, particles of the hexagonal Laves phase in the powders of Nb20 and Nb35 alloys were much harder to deform (Table 9). In consequence, the slightly increased porosity could be due to the presence of the pores within the phase’s interior or due to the barrier effect of the Laves phase particles.

**Figure 5:**
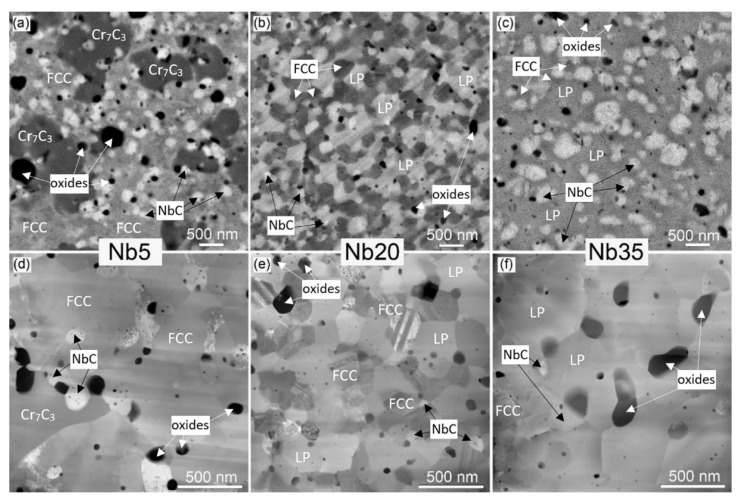
Electron microscopy micrographs of the CoCrFeNiNbX (X = 5, 20, 35 at.%) high-entropy alloys: (**a**–**c**) scanning electron microscopy—backscattered electron signal (SEM BSE), (**d**–**f**) scanning transmission electron microscopy, high angle annular dark field signal (STEM HAADF); LP marks Laves phase.

The microstructure of the alloys was examined by electron microscopy. From SEM backscattered (BSE) electron micrographs (Figure 5a–c) and STEM high-angle annular dark-field (HAADF) micrographs (Figure 5d–f), it is visible that the grains and phases in the alloys significantly differ. In the BSE or HAADF signal, the darkest, mostly round particles are oxides. The quantified microstructure parameters (size range) of all phases are summarized in Table 5.

**Figure 6:**
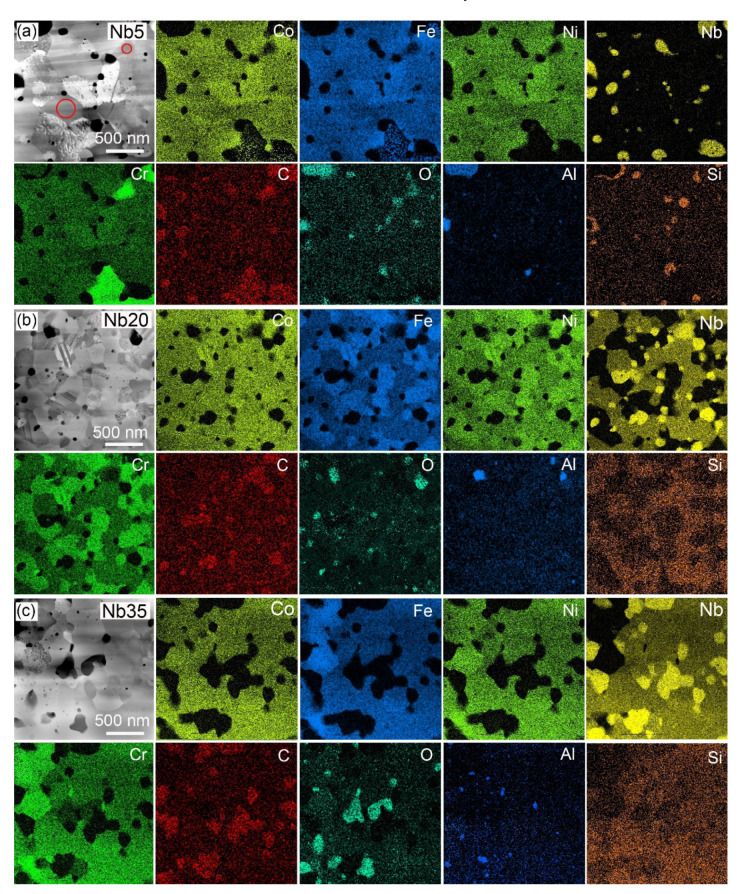
STEM high-angle annular dark-field (HAADF) micrographs and corresponding energy dispersive spectroscopy (EDS) maps of the Nb5 (**a**), Nb20 (**b**), and Nb35 (**c**) CoCrFeNiNbX high-entropy alloys. Red circles in the inset in the micrograph (**a**) demonstrate pop-up areas used for the quantification of the chemical composition of relevant phases from EDS map data cubes; the results are summarized in Table 6. At the request of the reviewer, the color levels indicating concentrations of Co, Fe, Cr, and O in the main phases were adjusted to better represent values in Table 6.

From the STEM EDS maps (Figure 6), it follows, that the biggest oxides in the Nb5 alloy are SiO_2_ particles (Figure 6a), and all alloys also contain large Al_2_O_3_ oxides (Figure 6). Al was not added; its presence—in a very small amount—is probably from impurities in the powders. In the Nb20 and Nb35 alloys, the XRD analysis detected ~1 wt.% of Nb_x_O_y_ oxides (Figure 5, Table 2), and some of them were found also on STEM EDS maps. The presence of SiO_2_ particles suggests in-situ reactions of the initially added SiC with the residual O forming a thermodynamically more stable SiO_2_ product. However, this reaction occurred only in the Nb5 alloy (Figure 6a). In Nb20 and Nb35 alloys, silicon is preferentially built-in in the Laves phase (marked LP in the figure), where it is uniformly distributed (Figure 6b,c). The Cr_7_C_3_ carbides appear in the BSE signal in Figure 5a in the second darkest grey level. They have an irregular shape and their size ranges from 0.5 to 1.5 μm (Figure 5 and Figure 6, Table 5). The majority FCC matrix of the Nb5 alloy, with grain size ranging from 100 to 750 nm (Figure 5a), is imaged in a medium grey level, while the NbC carbides (50–450 nm in size) containing heavy Nb are the lightest. Their size range (50–300 nm) is the narrowest of all studied alloys (Table 5).

In the Nb20 alloy (Figure 5b), the FCC matrix also appears in the medium grey level, yet somewhat darker than in Figure 5a; its grain size is somewhat finer (100–500 nm) than in the Nb5 alloy. The Laves phase (140–370 nm in size) contains about 30 at.% of Nb (Table 6) and so it appears in lighter grey, while NbC carbides (more than 70 at.% of Nb, 50–300 nm in size) are the lightest (Figure 5b).

In the Nb35 alloy (Figure 5c), the majority phase (74.2 wt.%—Table 2) is the medium grey Laves phase, with nearly the same grain size range (130 to 370 nm) as in the Nb 20 alloy. The minority FCC matrix (7.4 wt.%), having 100 to 350 nm grains, appears somewhat darker. The lightest particles are NbC carbides (50 to 600 nm in size) with a 17.4 wt.% fraction.

The STEM HAADF micrographs (Figure 5d–f) allow to distinguish very small oxide particles (from ~10 nm); furthermore, in the Nb20 alloy, they are also grown in twins, typical for FCC solid solutions (Figure 5e). On the other hand, the matrix, Cr_7_C_3_, and Laves phase particles are difficult to discern, since they similarly scatter fast electrons and so their contrasts merge (Figure 5d–f); nevertheless, in STEM EDS Nb maps (Figure 6), all these phases can be easily recognized because they have a distinctly different Nb content.

STEM EDS mapping enables obtaining not only a qualitative distribution of elements in the microstructure but also quantifying the composition of the microstructure components, i.e., the oxide and carbide particles, the Laves phase, and the adjacent CoCrFeNiNbX matrix. The average values, obtained from five measurements (pop-up areas marked in Figure 6a) on several EDS maps of the investigated alloys, are given in Table 6. From the quantification, it follows that chromium in the Cr_7_C_3_ carbides in the Nb5 alloy (Figure 6a) is substituted by 6 at.% of Fe and 3 at.% Co, while the content of Ni, Nb, and Si is only in the range of 0.4 to 0.6 at.% for each element. Hence, the carbide should be better designed as M_7_C_3_ (M = Cr, Co, Fe, Ni, Nb). On the other hand, in all presented alloys, niobium in NbC is substituted to a much lower extent, with the maximum amount of Co, Cr, and Fe in the range from 1.1 to 1.4 at.%, and the content of Ni, Si, and Al around 0.5 at.% for each. In the Nb5 alloy, the concentrations of Co, Fe, and Ni in the majority FCC matrix are similar to the nominal composition (Table 1), except for a somewhat lower content of Cr (14.7 at.%), and very low content of Nb (only 0.6 at.%) and Si (1.1 at.%). A relatively high concentration of carbon (7.8 at.%) in the FCC matrix is due to using n-heptane to avoid cold welding during mechanical alloying. Furthermore, in the Nb5 alloy, the maps also show Si and Al oxides (Figure 6a).

The Laves phase in the Nb20 and Nb35 alloys has a very similar composition; from the existent phases, it dissolves the highest amount of silicon (2.5 to 2.8 at.%), some oxygen (2.2 to 2.7 at.%) and carbon (1.5 to 2.3 at.%). The concentrations of Co, Cr, Fe, and Ni in the FCC matrix of the Nb20 and Nb35 alloys are not very different, except for the content of Nb, which is 2.0 and 3.6 at.%, respectively. The amounts of carbon (5.0 and 3.4 at.%) are somewhat lower than in the Nb5 alloy. From the comparison of the distributions of Al and O in Figure 6b,c, it follows that oxides dissimilar to alumina should be present. These are Cr_2_O_3_, which can be identified from the comparison of STEM HAADF micrographs with Cr and O distribution maps, and Nb oxides, identified from the comparison of Nb and oxygen maps. The Nb oxides form close to the NbC carbide particles (Figure 6b,c). It should be noted here that the maps of chromium and oxygen are superimposed to some extent because the Ka line of oxygen has an energy of 0.525 eV, which lies between the La of chromium (0.500 eV) and the L1 of chromium (0.573 eV). The chromium map is drawn using the Ka of chromium (5.411 eV), but the La and L1 of chromium contribute to the energy window used for oxygen mapping. In consequence, in the maps, oxygen is always in the same place as chromium (Figure 6). This is an artifact, which cannot be eliminated. Nevertheless, to bring the distribution of elements (Figure 6) and the composition list (Table 6) close together, several maps were modified. The intensities of the color representing O were generally suppressed and also locally reduced in the areas where O was not present (e.g., Cr_7_C_3_). Conversely, the Co and Fe intensities were increased in the Cr_7_C_3_ carbide area to better express the values of 3 and 6 at.%, respectively (Figure 6a). Similarly, in the Nb35 alloy, the green (Cr) and blue (Fe) colors were intensified in the FCC solid solution (Nb lean phase) to better distinguish the FCC phase Cr and Fe distribution from the surrounding Laves phase (Figure 6c). Consequently, the modified maps better represent the average quantified values.

The Laves phase has a Co_2_Nb-type crystal structure, with crystallographic parameters *a* = 0.47985 nm and *c* = 0.78675 nm, or *a* = 0.48033 nm and *c* = 0.78153 nm in Nb20 and Nb35 alloy, respectively. Jiang et al. [6] reported the composition of this Laves phase as Co(Ni,Fe,Cr)_2_Nb, while Liu et al. [24] reported a slightly different composition of (Co,Fe,Ni)_2_(Cr,Nb). Both compositions refer to HCP Laves crystallographic structures. Thus, according to STEM EDS analyses reported in Table 6, a Laves composition closer to Co(Ni,Fe,Cr)_2_Nb is supposed to be present in Nb20 and Nb35 alloys.

#### 3.1.2. Annealed Compacts

**Figure 7:**
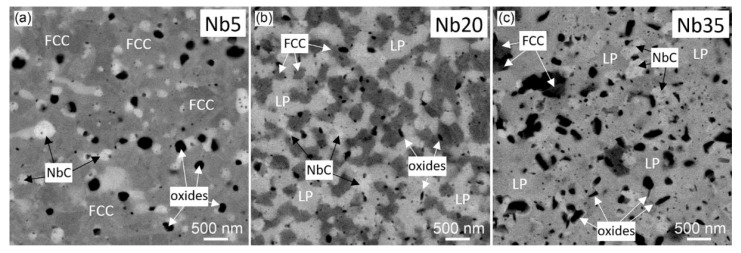
SEM BSE micrographs of the CoCrFeNiNbX (X = 5, 20, 35 at.%) high-entropy alloys after annealing for 100 h at 800 °C: (**a**) Nb5, (**b**) Nb20, (**c**) Nb35; LP marks Laves phase.

SEM-BSE micrographs of the compacts annealed for 100 h at 800 °C are in Figure 7. In comparison with the as-prepared alloys (Figure 5a–c), the microstructure apparently changed in the case of Nb5 and Nb35 alloys. After the long-term annealing of the Nb5 alloy at 800 °C, large particles of the Cr_7_C_3_ carbides disappeared. In the FCC matrix, there are only bright NbC carbides and dark oxides (Figure 6a). The grain and phase structure of the annealed Nb20 alloy at the SEM scale is practically the same as in the initial condition (Figure 5b); FCC matrix, NbC carbides, Laves phase, and oxide particles are uniformly distributed. The only difference is in the somewhat higher density of oxides, also having oblong or platelet form (Figure 7b). On the other hand, in the annealed Nb35 alloy containing the majority Laves phase, NbC carbides, and minority FCC phase, the number of oxides is significantly higher than in the as-compacted condition. Furthermore, these particles are much larger than in the initial condition, and they have frequently elongated shapes (Figure 7c).

**Figure 8:**
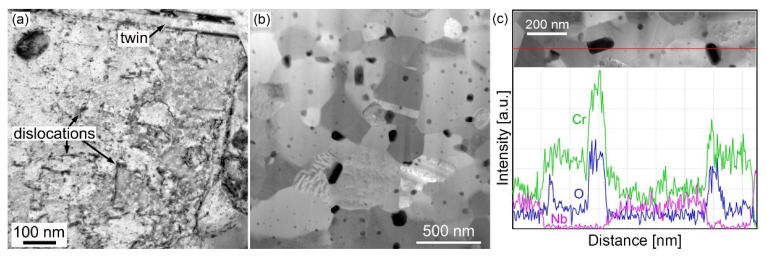
Fine microstructure of the CoCrFeNiNb20 high-entropy alloy annealed for 100 h at 800 °C: (**a**) Bright-field TEM image of an FCC matrix grain, (**b**) STEM HAADF micrograph, (**c**) STEM EDS line analysis across two oxide particles.

The microstructure of the annealed Nb20 alloy was also examined at a finer scale using transmission electron microscopy. Figure 8a shows a typical bright-field image of the annealed FCC matrix grain. After sintering at 1000 °C and long-term annealing at 800 °C, the substructure is recovered to a large extent and there is only a low density of dislocations and some annealing twins inside the FCC matrix grains. In contrast to the papers of Fan et al. [11], He et al. [25], or Cao et al. [26], who observed lamellar Mg_3_Cd-type Co-Ni-Nb rich τ-phase precipitating from the FCC solid solution of the CoCrFeNiNb_x_ matrix during annealing at 800 °C for 120 h, we did not find any precipitates. From STEM-HAADF imaging (Figure 8b), it follows that the long-term annealing at 800 °C led to coarsening of the oxide particles, which also appear in higher density in comparison with the as-compacted alloy (Figure 5e). The coarser particles often have an elongated or rectangular shape. An EDS line analysis across two particles in Figure 8c indicates that besides oxygen, these particles contain chromium or chromium and a minor content of niobium, respectively.

Results of the XRD phase analysis of the compacts annealed for 100 h at 800 °C are summarized in Table 7. The phase composition of the Nb5 alloy changed. The Cr_7_C_3_ carbide completely disappeared and a new oxide, Ni_6_Nb_6_O (JCPDS card no. 01-082-0946, space group Fd3m), was formed. The fraction of the FCC matrix increased from 75.7 to 88.1 wt.% and the amount of the NbC carbide decreased from 9.8 to 6.1 wt.%. Using the last-generation high-energy resolution 1Der detector enabled us to also distinguish a small amount (0.3 wt.%) of NbO_2_ (JCPDS card no. 01-082-1142, space group P42/mnm) and CrNbO_4_ oxides (JCPDS card no. 01-081-0909, space group P42/mnm). On the other hand, the long-term annealing at 800 °C did not lead to a different phase composition of the Nb20 alloy, if we do not take into account small amounts (0.1 to 0.4 wt.%) of oxides NbO_2_, Cr_2_O_3_ (JCPDS card no. 01-076-0147, space group R-3c), and CrNbO_4_, which were not distinguished by the less sensitive detector. However, there is a small change in the fraction of the main phases. The amount of the Laves phase increased from 34.5 to 39.1 wt.%, while the fractions of the FCC matrix and NbC carbide decreased from 51.9 to 48.9 wt.%, and from 12.6 to 11.5 wt.%, respectively. In the annealed Nb35 alloy, the fractions of the Laves phase and NbC carbide remained the same. A relatively high fraction (2.5 wt.%) of a new oxide CrNbO_4_ (JCPDS card no. 01-081-0909, space group P42/mnm) formed at the expense of the FCC matrix, and small amounts (0.1 to 0.2 wt.%) of NbO_2_ and Cr_2_O_3_ were also found.

Phase changes determined by XRD are in agreement with SEM and TEM observations which showed the vanishing of the Cr_7_C_3_ phase in the Nb5 alloy (Figure 7a), no qualitative change of the main phase constituents in the Nb20 alloy (Figure 7b and Figure 8b), and higher amount of oxides (identified mainly as CrNbO_4_) in Figure 7c. Taking into account high thermal stability of the Laves phase reported in Ref. [14] and also our results for the annealed Nb35 alloy, where the amount of the Laves phase and NbC carbide after long-term annealing at 800 °C remained the same, small differences in the fractions of main phases in the annealed Nb20 alloy can be assigned rather to local inhomogeneities in the samples than to phase transformations.

### 3.2. Mechanical Properties

#### 3.2.1. Hardness and Instrumented Microhardness Measurements

Results of hardness testing are summarized in Table 8, from which it follows that the Vickers hardness HV1, instrumented microhardness, and elastic modulus increase in an important manner with the Nb content. The hardness starts at 398 HV1 for the Nb5 alloy, raises to 798 HV1 for the Nb20 alloy, and up to 1114 HV1 for the Nb35 alloy. The observed behavior is in good agreement with the research of others [6,7]. The HV1 values are in a sort of good correlation with the instrumented HV_IT_. The slightly higher values measured by the instrumented method are caused by the difference in the evaluation of results. It should be noted that the HV1 is measured by the optical evaluation of the present imprint, while the instrumented HV_IT_ is determined from the unloading part of the depth–force curve. The increase in hardness and elastic modulus can be directly related to the increasing content of the HCP Laves phase, being 34.5 wt.% in the Nb20 alloy and 74.2 wt.% in the Nb35 alloy (Table 2).

**Figure 9:**
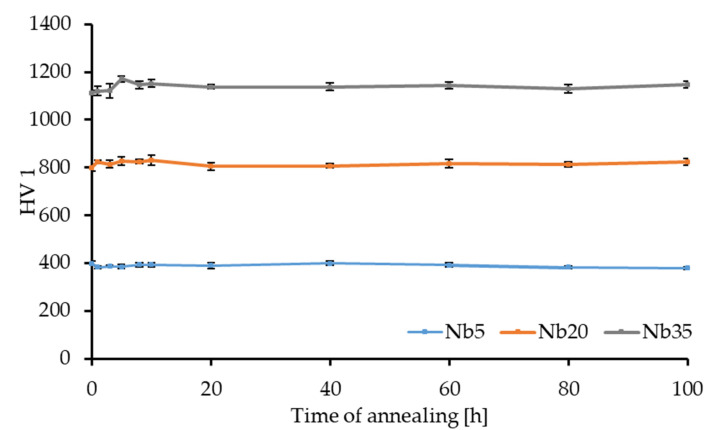
Thermal stability of the MA + SPS alloys during long-term annealing at 800 °C.

HV1 measurements were also used for testing the thermal stability of the compacts during long-term annealing. He et al. [7] reported an increase in the ductility of the arc-melted CoCrFeNiNb alloys when annealed at 750 °C. We have chosen the annealing temperature of 50 °C higher to avoid the microstructural coarsening at 900 °C reported by the same author. The plot in Figure 9 shows that at 800 °C all three alloys exhibit outstanding thermal stability since they maintained their hardness throughout the whole annealing period of 100 h. Such behavior can be expected since HEAs are in general known to have a low diffusivity of individual elements as one of their four core effects. In addition, the presence of the Laves phase in Nb20 and Nb 35 alloys is also beneficial since at 800 °C it is thermally stable [14].

#### 3.2.2. Compressive Deformation Tests

##### Laboratory Temperature Testing

**Figure 10:**
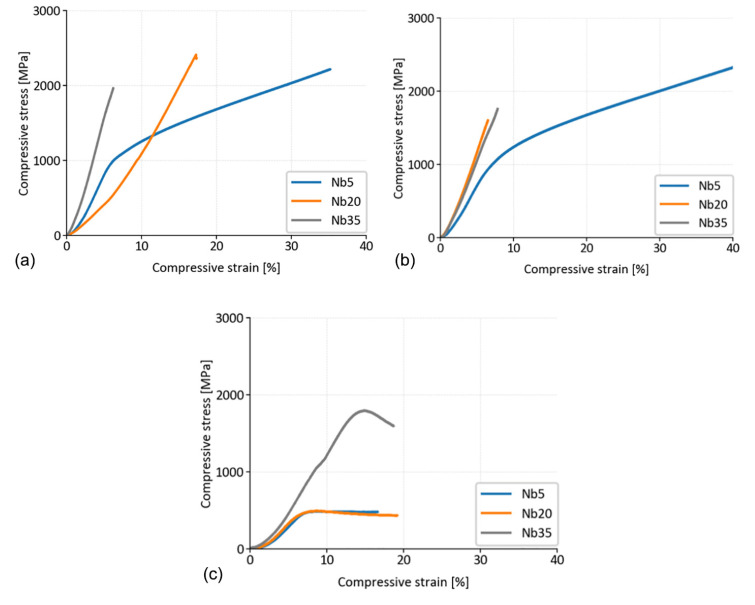
Compressive stress–strain diagrams of the MA + SPS CoCrFeNiNbX (X = 5, 20, 35 at.%) alloys: (**a**) in as-prepared state at LT, (**b**) at LT after annealing for 100 h at 800 °C, (**c**) at the temperature of 800 °C.

The compressive deformation tests were carried out at laboratory temperature (LT) in the as-prepared and annealed state (for 100 h at 800 °C) as well as at a temperature of 800 °C. Figure 10 shows the best stress–strain diagrams fitting the average values of compressive yield stress (CYS), and ultimate compressive strength (UCS), summarized in Table 9. The deformation behavior of the alloys at LT corresponds to the phase composition of the alloys (fraction of the ductile FCC matrix and hard but brittle HCP Laves phase) which is changing with the increasing Nb concentration (Table 2). The higher the content of the HCP Laves phase, the higher the strength of the alloy. However, this is also accompanied by increasing brittleness (Figure 10a,b). The UCS of the Nb35 alloy is lower than that of the Nb20 alloy because the Nb35 alloy is composed mainly of the brittle Laves phase and so the material fractures earlier in the elastic region. The Nb20 alloy contains about 52% of the plastic FCC phase and so it presents a lower Young modulus and does fracture in the elastic region, but later.

The Nb5 alloy does not contain any Laves phase and so it is very ductile, showing interesting work hardening ability and total strains > 35%, similar to the data in Ref. [5]. It is worth noticing that this material is very ductile although it contains a relatively high content of NbC (9.8 wt.%) and Cr_7_C_3_ (14.5 wt.%) carbides—in total the highest total content of carbides (24.3 wt.%) among all three alloys (Table 2). The CYS of the Nb5 alloy is quite high, reaching 953 MPa in the as-prepared state (Table 9).

With increasing the Nb content, the alloys completely lost their plasticity. They became brittle and fractured in the elastic region of the deformation diagram, showing scattered results of UCS, manifested by increasing values of the standard deviations (Table 9). The Nb20 alloy reached the highest UCS of 2413 MPa as has been published in our previous paper [23], and relatively good reproducibility of the results. This value is slightly higher than that of He et al. [7], reporting the increase in UCS up to 2392 MPa for the arc-melted CoCrFeNiNb_0.65_ being annealed for 24 h at 600 °C. Considering the amount of hard and brittle Laves phases, our MA + SPS CoCrFeNiNb20 alloy was showing only a slightly lower UCS than the arc-melted CoCrFeNiNb_0.8_ alloy (2479 MPa), as reported by Yu et al. [9]. Nevertheless, the chemical composition and the preparation route were different, and the CoCrFeNiNb_0.8_ alloy exhibited fairly decent plasticity associated with work hardening. Our CoCrFeNiNb35 (Nb35) alloy showed extreme brittleness, reducing its average UCS down to 1821 MPa. Such behavior can be attributed to the microstructural appearance, where the brittle HCP Laves phase has a majority (74.2 wt.%).

The annealing of the alloys for 100 h at 800 °C influenced the deformation behavior mainly in the case of the Nb20 and Nb35 alloys. While the CYS of the Nb5 alloy slightly decreased to 930 MPa and the material maintained its ductility (Figure 10b), the UCS of the Nb20 alloy lowered to 1518 MPa, and the UCS of the Nb35 alloy remained constant within the experimental error. However, the Nb20 and Nb35 alloys showed higher brittleness (Figure 10b), increasing the values of standard deviation in an important manner (Table 9). This behavior can be explained by microstructural changes. It was found that long-term annealing at 800 °C leads to the formation of CrNbO_4_ and Cr_2_O_3_ particles (Table 7, Figure 7 and Figure 8). Many of them are coarse and have an oblong or platelet form, increasing the stress intensity factor at the particle–matrix/Laves phase interfaces in the annealed alloys, already brittle in the as-compacted condition. In consequence, the UCS values of the annealed alloys Nb20 and Nb35 are more scattered due to easier crack formation in the elastic part of the stress–strain curves.

##### Testing at 800 °C

During the compression tests at an elevated temperature of 800 °C, the alloys responded differently. The Nb5 alloy composed of ~75 wt.% of FCC solid solution has a typical stress–strain curve of a dynamically recovering material. After the initial hardening, the Nb5 alloy is deformed at constant stress, indicating a dynamic equilibrium between multiplication and annihilation of dislocations. The value of CYS is relatively low (~450 MPa) and the value of UTS is not much different (~500 MPa). On the other hand, the Nb35 alloy contains only a small fraction (7.4 wt.%) of FCC solid solution embedded into the HCP Laves phase. Therefore, the influence of the FCC solid solution grains is limited and the behavior of the Nb35 alloy at 800 °C is dominated by the deformation of the hexagonal Laves phase through the activation of more slip systems at higher stresses [27]. In consequence, the material shows much higher values of CYS and UCS, 1695 and 1817 MPa, respectively.

Applying the rule of mixtures to the Nb20 alloy, containing 34.5 wt.% of Laves phase, one would expect CYS between the values of Nb5 and Nb35. However, repeating the experiments three times shows the same stress–strain curve as in the case of the Nb5 alloy (Figure 10, Table 9). The reason for this deformation behavior is the relatively slow strain rate (0.001 s^−1^) combined with a high temperature of 800 °C at which the dislocations in the FCC matrix are very mobile. In the FCC FeCoCrNi high-entropy alloy deformed at 800 °C, Lin et al. [28] observed a much lower density of dislocations than after deformation at 600 °C. The FCC matrix of our Nb20 alloy contains FeCoCrNi and only 2 at.% of Nb (STEM EDS analysis, Table 6), so we could expect similar dislocation mobility. The Nb20 alloy contains ~52 wt.% of the soft FCC matrix (Table 2) which is uniformly distributed, often forming continuous matrix channels ~500 nm wide (Table 5, Figure 5b). According to the strengthening model for a CoCrFeNiNb high-entropy alloy at laboratory temperature (LT) by Průša et al. [23], the Laves phase particles could strengthen the alloy by three mechanisms: (i) by Orowan-type dislocation looping and bypass mechanism, (ii) by the thermal expansion coefficient difference between the matrix and the Laves phase, and (iii) by the load transfer contribution from the matrix to the Laves phase. As the Laves phase particles are along the FCC matrix grain boundaries (Figure 5e), the dislocations gliding at LT would rather contribute to thickening the FCC grain boundaries rather than bypassing the Laves phase particles. Therefore, this strengthening contribution is considered by Průša et al. [23] as not significant at LT, because the mean distance between the Laves particles (which is of the order of the FCC grain size of ~500 nm) is relatively large. At 800 °C, this contribution will also not be significant; this is also the case for the impact of the thermal expansion coefficient difference (because of the presence of a soft FCC matrix). So, we must only take into account the load transfer mechanism from the FCC matrix to the Laves phase. However, at 800 °C, as already mentioned above for the Nb5 alloy, the FCC matrix is deformed at constant stress, indicating a dynamic equilibrium between multiplication and annihilation of dislocations. The load transfer from the matrix to the Laves phase is thus negligible because the gliding dislocations often annihilate before reaching the obstacles and the deformation behavior of the Nb20 alloy is thus controlled by the soft FCC phase. In consequence, the stress–strain curve is the same as in the case of the Nb5 alloy.

## 4. Conclusions

CoCrFeNiNbX (X = 5, 20, and 35 at.%) high-entropy alloys with an addition of 5 at.% of SiC were prepared by mechanical alloying (MA) and spark plasma sintering (SPS) to generate materials with submicrometric grains. The FCC high-entropy alloys, NbC carbides, and hexagonal Laves phase had already formed during MA. In the Nb5 alloy, the SPS compacting led to the formation of Cr_7_C_3_ carbides, and NbO_2_, SiO_2,_ and Al_2_O_3_ oxidic particles. The added SiC underwent an in-situ reaction transforming it into thermodynamically more stable SiO_2_ only in the Nb5 alloy, while in the Nb20 and Nb35 alloys, silicon dissolved in the present phases. The fraction of the FCC solid solution decreased with increasing Nb concentration at the expense of the NbC carbide and the Laves phase. Long-term annealing at 800 °C led to the disappearance of the Cr_7_C_3_ carbide in the Nb5 alloy, and new oxides—Ni_6_Nb_6_O, Cr_2_O_3_, and CrNbO_4_—were formed.

The compression deformation behavior depended on the fraction of the ductile FCC solid solution and hard but brittle hexagonal Laves phase. At laboratory temperature, the Nb5 alloy, containing only FCC matrix and carbide particles, was relatively strong and very ductile. At higher Nb content (Nb20 and Nb35), the alloys became brittle. The annealing of the alloys for 100 h at 800 °C influenced the deformation behavior of the alloys only a little. The CYS and UCS values lowered or remained constant within the experimental scatter. The Nb5 alloy conserved its plasticity and the Nb20 and Nb35 alloys maintained or even increased their brittleness. At 800 °C, the Nb5 and Nb20 alloys deformed almost identically (CYS ~450 MPa, UTS ~500 MPa, plasticity ~18%), whereas the Nb35 alloy was much stronger (CYS of 1695 MPa, UCS of 1817 MPa) and preserved comparable plasticity.

## Figures and Tables

**Table 1:** Chemical composition of the investigated compacts determined by XRF (at.%).

Alloy	Co	Cr	Fe	Ni	Nb	Si
CoCrFeNiNb5	20.31	22.00	28.18	21.47	5.77	2.27
CoCrFeNiNb20	17.44	19.68	22.32	18.65	19.70	2.21
CoCrFeNiNb35	13.76	15.42	20.21	14.83	32.72	3.06

**Table 2:** Results of the X-ray diffraction (XRD) phase analysis (wt.%) *.

Alloy	FCC Matrix	NbC	Cr_7_C_3_	Laves Phase	Nb_x_O_y_
CoCrFeNiNb5	75.7 ± 0.1	9.8 ± 0.2	14.5 ± 0.7	-	-
CoCrFeNiNb20	51.9 ± 0.2	12.6 ± 0.1	-	34.5 ± 0.2	1.0 ± 0.5
CoCrFeNiNb35	7.4 ± 0.4	17.4 ± 0.2	-	74.2 ± 0.1	0.9 ± 0.5

* The mentioned errors follow from the Rietveld method, i.e., calculation errors. Nevertheless, it is generally known that the errors of the major and minor phases for powders do not exceed 1.5 and 0.5 wt.%, respectively.

**Table 3:** Crystal lattice parameters determined by XRD (nm).

Alloy	FCC Matrix	NbC	Cr_7_C_3_	Laves Phase
CoCrFeNiNb5	*a* = 0.35789 (0)	*a* = 0.44689 (0)	*a* = 0.70062 (7) *b* = 1.23547 (2) *c* = 0.45153 (0)	-
CoCrFeNiNb20	*a* = 0.35918 (3)	*a* = 0.44531 (4)	-	*a* = 0.47989 (5) *c* = 0.78075 (8)
CoCrFeNiNb35	*a* = 0.35885 (5)	*a* = 0.44503 (2)	-	*a* = 0.48033 (4) *c* = 0.78153 (1)

**Table 4:** The average surface porosity of the polished compacts assessed using the threshold method. (* Average surface porosity determined in our previous work [23]).

Alloy	CoCrFeNiNb5	CoCrFeNiNb20 *	CoCrFeNiNb35
Area fraction [%]	0.03 ± 0.01	0.23 ± 0.14	0.25 ± 0.11

**Table 5:** FCC matrix, carbide, Laves phase, and oxide particle size range determined from SEM, STEM, and STEM EDS micrographs (nm).

Alloy	FCC Matrix	NbC	Cr_7_C_3_	Laves Phase	Oxides
CoCrFeNiNb5	100–750	50–450	500–1500	-	10–500
CoCrFeNiNb20	100–500	50–300	-	140–370	10–300
CoCrFeNiNb35	100–350	50–600	-	130–370	10–350

**Table 6:** The average composition of carbide particles, Laves phase, and FCC solid solution (matrix) from EDS maps (at.%). (Five readouts from several EDS maps, not only from Figure 6).

Alloy	Co	Cr	Fe	Ni	Nb	Si	Al	C	O
**Nb5**									
Cr_7_C_3_	3.0 ± 1.6	52.2 ± 2.6	6.1 ± 1.1	0.4 ± 0.6	0.6 ± 0.3	0.6 ± 0.2	-	36.7 ± 2.5	-
NbC	1.4 ± 0.3	1.2 ± 0.4	1.3 ± 0.4	0.1 ± 0.3	65.5 ± 12.6	0.4 ± 0.5	0.3 ± 0.2	24.1 ± 7.8	5.5 ± 2.4
matrix	23.2 ± 0.3	14.7 ± 0.7	28.7 ± 0.7	23.2 ± 0.2	0.6 ± 0.3	1.1 ± 0.1	0.1 ± 0.1	7.8 ± 1.6	0.5 ± 0.6
**Nb20**									
NbC	1.3 ± 0.6	1.3 ± 1.0	1.2 ± 0.4	–	74.6 ± 7.3	1.7 ± 2.8	0.3 ± 0.4	14.3 ± 7.8	6.3 ± 3.7
Laves ph.	18.9 ± 1.7	11.4 ± 0.5	17.8 ± 4.5	14.5 ± 1.0	31.5 ± 1.2	2.8 ± 0.4	0.1 ± 0.1	2.3 ± 2.0	2.7 ± 3.1
matrix	19.1 ± 1.6	24.9 ± 1.4	26.5 ± 1.2	21.7 ± 1.2	2.0 ± 0.3	0.4 ± 0.3	0.1 ± 0.2	5.0 ± 2.8	-
**Nb35**									
NbC	1.1 ± 0.7	1.2 ± 0.3	1.1 ± 0.3	0.5 ± 0.4	72.2 ± 4.5	0.6 ± 0.3	0.4 ± 0.2	17.5 ± 5.2	5.3 ± 0.7
Laves ph.	14.7 ± 0.6	13.0 ± 0.8	17.8 ± 0.6	14.0 ± 0.5	33.9 ± 1.7	2.5 ± 0.2	0.3 ± 0.2	1.5 ± 1.6	2.2 ± 1.8
matrix	16.0 ± 0.6	24.7 ± 0.6	29.9 ± 1.4	21.5 ± 1.2	3.6 ± 0.8	0.4 ± 0.3	0.3 ± 0.2	3.4 ± 2.1	-

**Table 7:** X-ray diffraction (XRD) phase analysis after annealing for 100 h at 800 °C (wt.%).

Alloy	FCC Matrix	NbC	Ni_6_Nb_6_O	Laves Phase	NbO_2_	Cr_2_O_3_	CrNbO_4_
CoCrFeNiNb5	88.1 ± 0.4	6.1 ± 0.1	5.2 ± 1.2	-	0.3 ± 0.2	-	0.3 ± 0.2
CoCrFeNiNb20	48.9 ± 0.1	11.5 ± 0.2	-	39.1 ± 0.2	0.4 ± 0.2	0.1 ± 0.0	0.1 ± 0.1
CoCrFeNiNb35	5.2 ± 0.2	17.4 ± 0.2	-	74.6 ± 0.1	0.1 ± 0.0	0.2 ± 0.0	2.5 ± 0.2

**Table 8:** Vickers hardness HV 1, instrumented (IT) Vickers microhardness, and elastic modulus of studied alloys.

Alloy	CoCrFeNiNb5	CoCrFeNiNb20	CoCrFeNiNb35
HV 1	398.4 ± 9.0	798.1 ± 14.0	1113.7 ± 11.6
HV_IT_	467.4 ± 10.2	880.2 ± 27.4	1309.8 ± 64.3
E_IT_ [GPa]	208.4 ± 8.2	242.0 ± 11.1	259.1 ± 6.5

**Table 9:** Summary of the average compressive yield stress (CYS) and ultimate compressive strength (UCS) values [MPa] of studied alloys.

Alloy	LT		LT after 100 h at 800 °C		at 800 °C	
	CYS	UCS	CYS	UCS	CYS	UCS
CoCrFeNiNb5	953 ± 12	*	930 ± 16	*	453 ± 27	492 ± 18
CoCrFeNiNb20	**	2413 ± 89	**	1518 ± 276	442 ± 17	503 ± 13
CoCrFeNiNb35	**	1821 ± 144	**	1843 ± 468	1695 ± 110	1817 ± 109

* UCS could not be determined due to continuous work hardening of the material. ** CYS could not be determined due to the brittle behavior of the alloy.

## Data Availability

Data are contained within the article.
